# A deep learning model for the classification of atrial fibrillation in critically ill patients

**DOI:** 10.1186/s40635-022-00490-3

**Published:** 2023-01-13

**Authors:** Brian Chen, David M. Maslove, Jeffrey D. Curran, Alexander Hamilton, Philip R. Laird, Parvin Mousavi, Stephanie Sibley

**Affiliations:** 1 School of Computing, Queen’s University, Kingston, Canada; 2 Department of Critical Care Medicine, Queen’s University, 76 Stuart Street, Kingston, ON K7L 2V7 Canada; 3 Centre for Health Innovation, Queen’s University, Kingston, Canada

**Keywords:** Atrial fibrillation, Deep learning, Critical care

## Abstract

**Background:**

Atrial fibrillation (AF) is the most common cardiac arrhythmia in the intensive care unit and is associated with increased morbidity and mortality. New-onset atrial fibrillation (NOAF) is often initially paroxysmal and fleeting, making it difficult to diagnose, and therefore difficult to understand the true burden of disease. Automated algorithms to detect AF in the ICU have been advocated as a means to better quantify its true burden.

**Results:**

We used a publicly available 12-lead ECG dataset to train a deep learning model for the classification of AF. We then conducted an external independent validation of the model using continuous telemetry data from 984 critically ill patients collected in our institutional database. Performance metrics were stratified by signal quality, classified as either clean or noisy. The deep learning model was able to classify AF with an overall sensitivity of 84%, specificity of 89%, positive predictive value (PPV) of 55%, and negative predictive value of 97%. Performance was improved in clean data as compared to noisy data, most notably with respect to PPV and specificity.

**Conclusions:**

This model demonstrates that computational detection of AF is currently feasible and effective. This approach stands to improve the efficiency of retrospective and prospective research into AF in the ICU by automating AF detection, and enabling precise quantification of overall AF burden.

**Supplementary Information:**

The online version contains supplementary material available at 10.1186/s40635-022-00490-3.

## Background

New-onset atrial fibrillation (NOAF) is the most common cardiac dysrhythmia in critically ill patients with a reported incidence as high as 46% [1]. It is most often described in patients with sepsis with an incidence of 10–40% [2], but is seen in a variety of illnesses such as acute respiratory distress syndrome [3], non-cardiac thoracic surgery [4, 5], and trauma [6]. Critical illness is an independent driver of NOAF due to arrhythmogenic triggers such as electrolyte disorders, vasoactive medications, fluid overload and hypoxia [7]. Acute events during critical illness (e.g., infection, ischemia) accelerate cardiac remodeling and fibrosis, begetting further arrhythmias [8]. NOAF is independently associated with prolonged duration of hospital stay [2, 9] and significant morbidity and mortality [10]. Patients with NOAF are at an increased risk for in-hospital ischemic stroke [11] and 18% of patients who develop NOAF are discharged from the intensive care unit (ICU) with ongoing atrial fibrillation (AF) [12]. Patients who develop NOAF in the ICU may be at increased risk of having recurrent AF in the future and suffer the consequences associated with it, such as stroke and heart failure [13].

Given the consequentiality of developing NOAF, prevention and treatment of this arrhythmia are of great potential benefit. Unfortunately, NOAF is difficult to study, as the ability to detect it in real time and quantify its burden is limited in most ICU settings. There is difficulty capturing the arrhythmia in patients who develop paroxysmal atrial fibrillation as fleeting arrhythmias are often missed on telemetry or ECG [14]. Most studies of NOAF are retrospective and are based on diagnostic codes in large databases which rely on the accuracy and completeness of those codes for identification of patients and their long-term outcomes.

Development of detection algorithms to identify patients with NOAF would allow for precise estimates of the incidence and prevalence and inform future studies of NOAF in a critical care setting. Automated monitoring algorithms for the detection of AF have previously been developed using heart rate variability techniques [15, 16], however their application to continuous, high-frequency ECG data collected in ICUs by bedside monitoring is limited. In this paper, we describe the development and validation of a deep learning model to classify AF in a large database of continuous telemetry data collected on critically ill patients.

## Methods

**Fig. 1:**
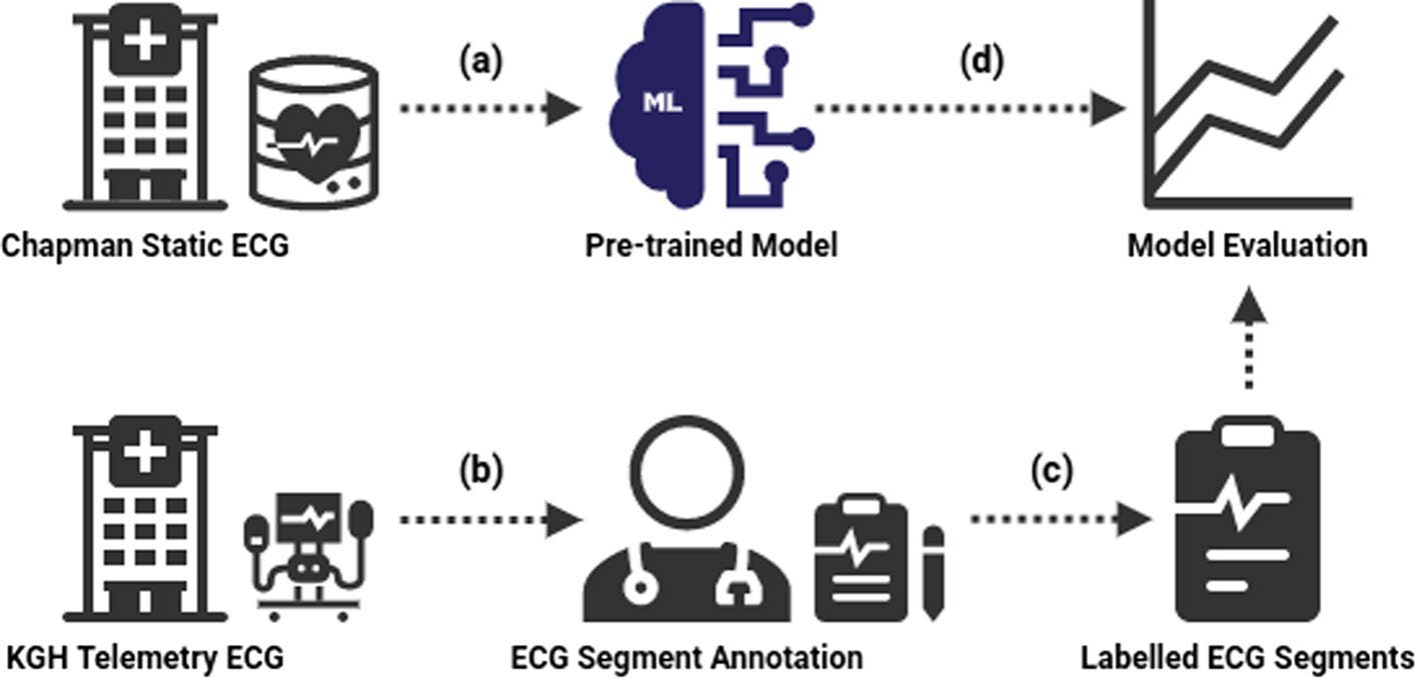
Summary of the experimental process. A deep learning model was trained on an external ECG dataset (**a**). ECG segments were extracted from our KGH database (**b**) and underwent expert annotation (**c**). The pre-trained model was evaluated as an AF classifier against our labeled data (**d**)

Figure 1 shows a high-level overview of our experimental process.

### Institutional dataset creation

The Queen’s University Health Sciences Research Ethics Board approved this study (File No: 6024689). The need for informed consent was waved because the data were collected as part of routine care and were stored in a de-identified format. Our main source of test data were collected from bedside monitors in the ICU at Kingston Health Sciences Centre (KHSC) in Kingston, Ontario, Canada, a 33-bed mixed-use medical-surgical, neurological, and trauma ICU. Both physiological waveforms and vital sign time series were collected from GE Solar monitors using Bedmaster software (Hill-Rom Holdings Inc, Chicago, Ill). These were stored in Critical Care Data Exchange Format [17] files for ease of querying and long-term archiving. Over 11TB of data were collected between 2015 and 2020, including oxygen saturation, arterial line, and ECG waveforms. ECG signals were sampled at 240 Hz and included leads I, II, III and V1.

### Model development

Building on our previous work [18], we used the publicly available Chapman 12-lead ECG dataset [19] for model development and training. This dataset consists of static 12-lead, 10 s ECG recordings from over 10,000 patients from Shaoxing People’s Hospital in Zhejiang, China. Leads I, II, III and V1 were extracted and the recorded segments were resampled from 500 to 240 Hz in order to match the dimensions of our institutional database at Kingston Health Sciences Center (KHSC). To translate between the KHSC classes and the Chapman diagnostic classes, we mapped the sinus bradycardia, sinus rhythm, sinus tachycardia and sinus irregularity classes to our sinus rhythm class, and the AF and atrial flutter classes to our AF class. We split the ECG segments with a 70/10/20% training/validation/testing split, stratified by rhythm type and patient gender.

### Model architecture and training

A deep convolutional neural network [20] was chosen as our ECG classification model. This specific classifier [21] was derived from an existing architecture shown to have state-of-the-art performance for AF detection on single-lead, portable device ECG [22]. The base architecture was kept as-is, with the exception of changes to the input dimensions to accommodate our 4-lead KHSC data.

Once initialized, the model was trained on the Chapman training dataset. Hyperparameter tuning was conducted on the Chapman validation set to select a high-performing configuration, and the final model was evaluated on the Chapman test set to ensure performance was comparable to the results found in the study by Zheng et al. [19]. A full breakdown of the model architecture and training parameters is found in Additional file 1: Fig. S4. Model development was conducted using the PyTorch Python Library [23].

To create a retrospective patient cohort, we selected files from the KHSC institutional dataset that could be directly linked back to a known patient admission and stay. Because patients could be moved between different beds in the ICU over the course of their admission, there was a risk of a particular recording containing waveform data for multiple patients if monitors were not properly re-configured after a move. To that end, we excluded any stays that had a bed move to ensure a one-to-one correspondence between the patients and the data files. This strict matching also ensured that admission and discharge times could be aligned reliably. Further filtering was conducted to ensure a sufficient quantity of ECG signal was present in each patient’s recorded waveforms. We excluded any stays that did not have all four ECG leads recorded. We removed cases with less than 10 s of continuous, unbroken ECG data. For the remaining records, we randomly sampled a 10-s segment of continuous ECG signal for each patient to create our KHSC dataset.

To obtain ground truth labels for the segments, we defined a set of eight disjoint classes for annotation (Table 1). These included seven categories of rhythms and a catch-all “Abstain” class for records deemed too noisy or possessing insufficient clear signal to assign a label. Annotation was conducted by two critical care physicians experienced in ECG interpretation and identifying arrhythmias present in an ICU context. A third critical care physician acted as a tiebreaker in cases of disagreements, and if all three reviewers disagreed the third reviewer’s label was used. Because not all classes defined prior to annotation were present in the labeled dataset, only those with greater than ten samples were included for further analysis. A second round of annotation was conducted with a critical care physician to collect additional information on potentially noisy segments. This involved a simple binary label indicating whether each segment was "clean" or contained a significant amount of noise. Clean samples had no baseline wander, motion artifact or signal drop off. Samples labeled “noisy” could still be categorized if the rhythm was interpretable and were labeled “abstain” if the signal was too noisy to determine the underlying rhythm. Free text comments were also collected about specific types of noise present for each noisy sample.

**Table 1:** Distribution of segments across all ECG rhythm classes in the annotated KGH data and the number of samples identified as noisy

Class	Count	Noisy
Sinus rhythm	746	127
Atrial fibrillation/atrial flutter	123	44
Pacemaker	20	5
Bigeminy and trigeminy	3	1
Ventricular tachycardia/fibrillation	0	0
Other tachyarrythmia cardia	0	0
Other bradyarrythmia	5	1
Non-diagnostic (abstain)	87	85

### Model evaluation

Due to the low expected prevalence of arrhythmias other than AF, we retained only segments labeled as sinus rhythm (SR) or AF from the KHSC dataset to test the pre-trained model. A standard set of binary classification metrics were employed: area under the receiver operator curve (AUROC), precision/positive predictive value (PPV), negative predicative value (NPV), specificity and recall/sensitivity. Segments with an AF label were considered as a positive test, while segments with a label of sinus rhythm were considered a negative test. In addition to evaluating overall performance on this subset of KHSC data, we derived independent performance measurements for segments that were identified as clean or noisy.

## Results

### Curation of local dataset

**Fig. 2:**
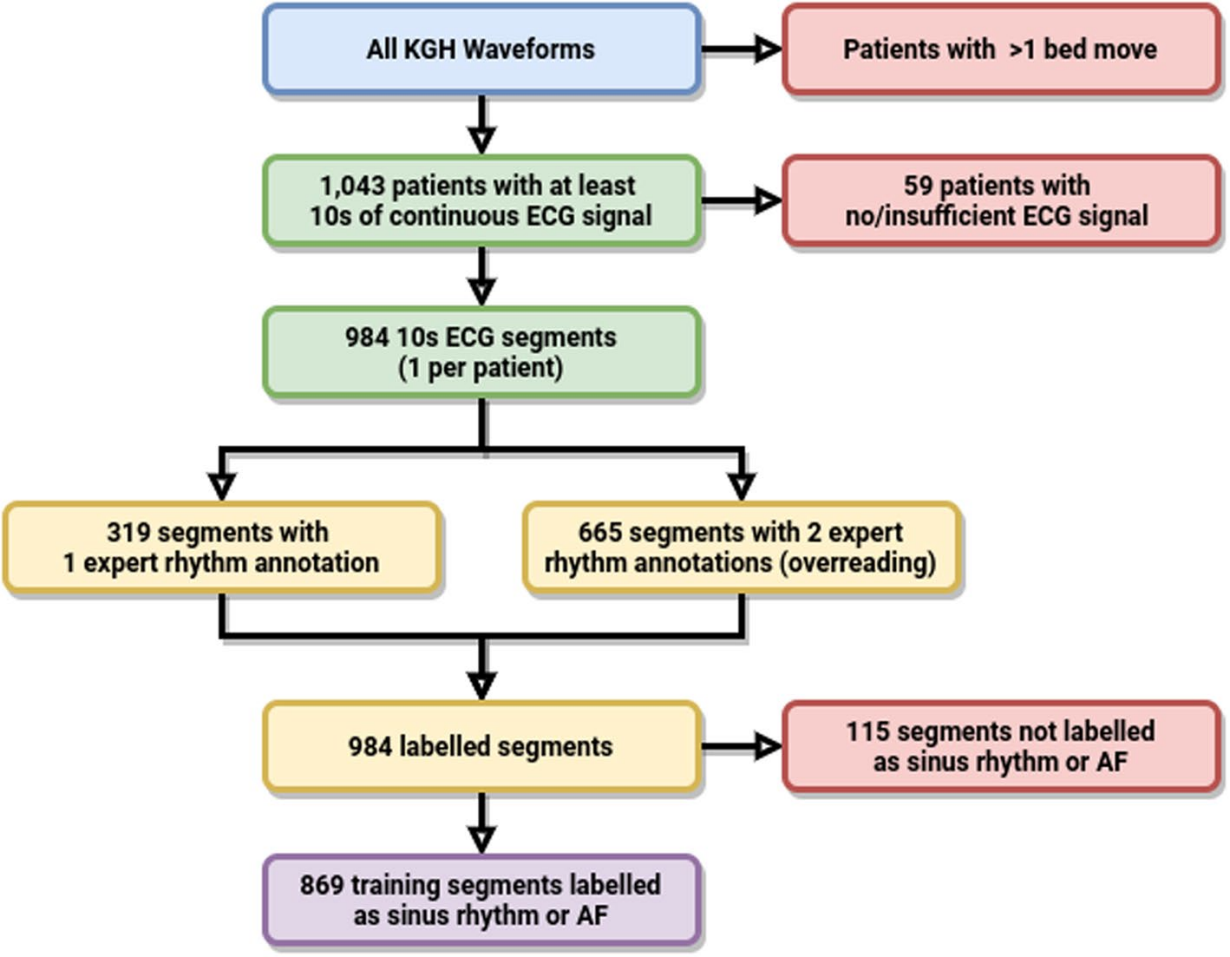
Flowchart for the patient cohort selection and data annotation process

In the KHSC dataset, a total of 1043 patients were found to have an ICU stay with no bed moves. Of these stays, 59 did not contain all 4 leads or a minimum of 10 s of continuous ECG signal. The median recording duration of the remaining 984 patient waveform records before sampling was 11.9 h. 665 were annotated by two critical care physicians, with a kappa of 0.81. The reviewers disagreed on 47 segments, of which 17 were labeled “abstain” by at least one reviewer. All three reviewers disagreed on 9 segments of which 5 were labeled “abstain” by the third reviewer. Given the good agreement between physicians, an additional 319 samples were annotated by the first critical care physician. All 984 segments received noise annotations. A flowchart summary of the sampling and annotation process may be found in Fig. 2.

Table 1 shows a breakdown of class counts and identified noise levels for the KGH data. Of the ECG segments annotated, the majority class was sinus rhythm and the majority arrhythmia was AF at 12.5%.

The sinus rhythm class contains all sinus rhythms regardless of rate, including sinus tachycardia and sinus bradycardia. No segments were labeled as ventricular tachycardia/fibrillation or other types of tachy/bradycardia.

Twenty-seven percent of records were found to contain some amount of noise, whereas segments in the non-diagnostic class were found to be almost completely noisy. Upon review of the segments in this class that were not labeled as noisy, we found that the discrepancy could be explained by disagreements during overreading. The AF class had the next highest noise level at 36%, more than twice that of sinus rhythm. Comments left for noisy segments fell into one of three categories: baseline signal artifact, baseline wander, and other unspecified artifacts. Representative examples for clean and each category of noise and counts of each type of noise may be found in Additional file 1: Table S3 and Additional file 1: Fig. S5.

After removing 115 segments not labeled as either SR or AF, we were left with a final testing dataset of 869 segments. The prevalence of positive AF samples in this subset was 14.1%, while 19.7% of samples were identified as noisy.

### Model performance

**Fig. 3:**
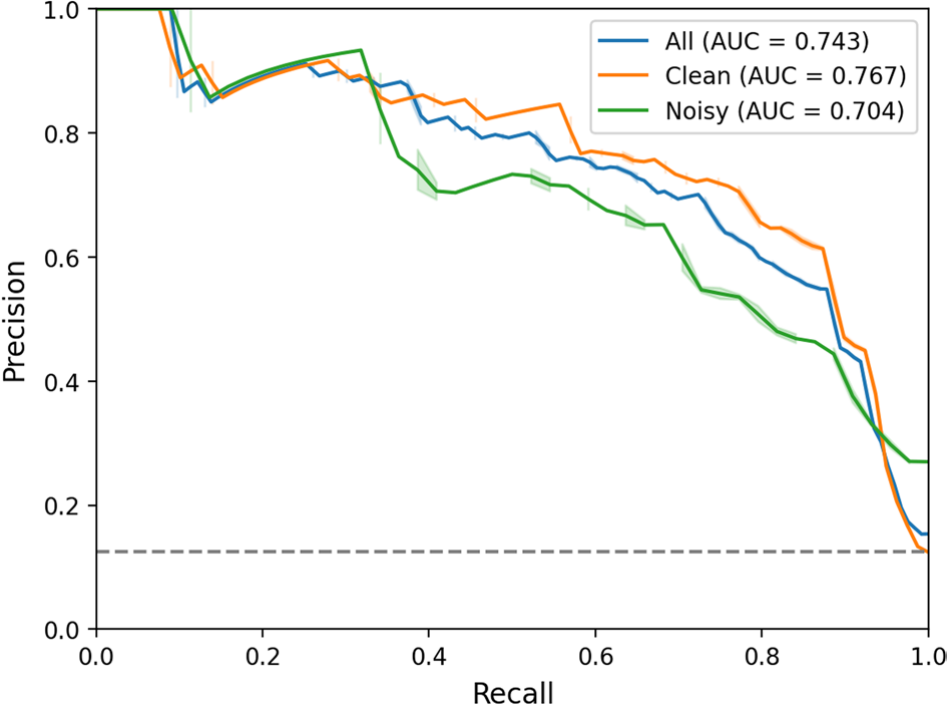
Precision–recall curves for the deep learning model on clean, noisy, and all labeled sinus and AF samples from the KGH dataset. The dashed gray line represents the expected performance of a random baseline classifier, i.e., the prevalence of the positive (AF) class

In the test set portion of the Chapman dataset, the deep learning model for binary classification of AF (positive) versus SR (negative) achieved an AUROC of 0.985, PPV of 0.828, sensitivity of 0.950 and specificity of 0.940 (Additional file 1: Fig. S6). The prevalence of the AF class in this test set was 23.5%, compared to 36.5% across the entire Chapman dataset. Model performance in the KHSC dataset is summarized in Table 2. Overall, the classifier demonstrated high sensitivity and specificity in identifying AF, with better performance seen among the clean segments compared to the noisy segments. Full precision–recall curves of KHSC data may be found in Fig. 3.

**Table 2:** Metrics for AF (test positive) versus sinus rhythm (test negative) classification on the KGH dataset. Results are shown for both the combined class samples and separately for clean and noisy subsets

Dataset	AUROC	PPV	NPV	Sensitivity	Specificity
All	0.935	0.548	0.971	0.837	0.886
Clean	0.948	0.623	0.978	0.835	0.935
Noisy	0.856	0.451	0.921	0.841	0.646

## Discussion

In this study, we applied a deep learning model for the classification of AF adapted from static 12-lead ECGs, to continuous telemetry data collected from an ICU. Despite training on a different type of ECG data, the model was highly sensitive and specific, with performance in clean data being better than that in noisy data.

Prior studies on AF detection from ICU telemetry data have been developed using continuous ECG telemetry waveform data from the Medical Information Mart for Intensive Care (MIMIC III) data set, and have primarily focused on engineered features derived from waveform characteristics [16, 24, 25]. In the study by Walkey et al. telemetry data from three cohorts of 50 patients was analyzed for interpretable signal using automated signal and noise detection. Two-minute segments were then assessed using an algorithm that detects R-waves using variable-frequency complex demodulation-based ECG reconstruction that evaluates the sample entropy, which would be expected to be higher in AF samples than in normal sinus rhythm. To differentiate premature atrial and ventricular beats, Poincaré plots and p-wave detection algorithms were used. When only signal and noise detection and R–R interval indices were used the algorithm had a sensitivity of 91%, a specificity of 71%, a PPV of 48%, a NPV of 96% and an accuracy of 76%. This improved with the addition of Poincaré plots and P-wave indices to a sensitivity of 100%, a specificity of 95%, a PPV of 85%, a NPV of 100% and an accuracy of 96% [25]. Similarly, Bashar et al. analyzed 198 septic subjects from the MIMIC III database using a heart rate variability algorithm using statistical parameters such as the root mean square of successive differences, Shannon entropy, and sample entropy, followed by Poincaré plots for premature atrial contraction/premature ventricular contraction (PAC/PVC) detection. The samples included 50 patients in a training set and 49 patients in a validation set, of which 11 were in AF. The sensitivity, specificity, accuracy, PPV and NPV were 100%, 94.74%, 95.92%, 84.62%, and 100%, respectively. The algorithm was tested on an additional 49 patients whose ECGs did not contain any PAC/PVC and a second test set of 50 patients where 25 patients had AF and 25 had NSR with PAC/PVCs. The combined test sets of 99 patients had a sensitivity, specificity, accuracy, PPV and NPV of 100%, 98%, 98.99%, 98%, and 100% [16].

In contrast to these studies, we have applied our model to a new data set from our institution that is more recent than the MIMIC III dataset. We have used a larger sample size than previous studies and have the added benefit of deploying the algorithm on raw data without any computationally expensive pre-processing. Our prevalence-dependent performance metrics (PPV) are more realistic given the class imbalance in our data (14.1% AF), which is in keeping with estimated prevalence of atrial fibrillation in ICUs, compared to the 50% AF prevalence in test sets in other studies [16, 24].

Several traditional learning models for the detection of atrial fibrillation have been published and are generally based on the mathematical characteristics of waveform analysis and typically require manual feature selection, extraction and classification. These extracted features may perform well in the data set in which they are developed, but require assumptions that may limit their reliability and their generalizability is uncertain. The advantage of a deep learning model is twofold: the selection and extraction features are built into the model, thus eliminating the need for separate steps, and the model is able to “learn” as more data are presented to it, with potential for model performance to be continuously improved [26]. Although deep learning approaches have been explored for static ECG [27], end-to-end deep learning approaches have seen very limited uptake for streaming data from ICU bedside monitors [28]. Transfer learning (training a model on one dataset and fine-tuning on a much smaller dataset before evaluating) has also been evaluated as a method for developing AF classification models when only limited annotated ECG data are available locally [29]. Building on this, we demonstrate that a deep learning model trained on static ECGs can identify AF in telemetry data, and expect that the performance of this model will improve with continued training.

Diagnosis of NOAF and inclusion into studies of this arrhythmia has traditionally relied on manual interpretation of ECGs, recordings of observed heart rhythms by bedside nurses, and billing codes (such as ICD-9 codes). Study of NOAF is further limited by a lack of common definition of the condition, with studies reporting different heart rates, durations of NOAF, and criteria for “new-onset” [30]. Use of automated algorithms could revolutionize the study of NOAF in critically ill patients. While heart rate variability algorithms require clean ECG samples of sufficient duration to reliably calculate statistical parameters, our machine learning approach allows the use of 10-s segments of telemetry data which are far less cumbersome to manually annotate and have the additional benefit of granularity that allows capture of very brief periods of NOAF that may be otherwise missed given the paroxysmal nature of the arrhythmia. This would aid in quantifying the burden of NOAF that is clinically important and contribute to a uniform definition of NOAF to be used in future studies. Continuous monitoring with computational classification may enable prediction of the development of atrial fibrillation [31] and enable the stratification of patients with varied duration of NOAF, potentially allowing predictive enrichment in clinical trials. Clinically, enhanced detection of these arrhythmias has the potential to markedly enhance our understanding of the true burden of disease and management of this arrhythmia in critically ill patients. Real-time identification of NOAF would enable immediate notification of practitioners and rapid administration of treatment if needed. This study provides the ground work for development of predictive models, allowing for identification of patients at higher risk of developing the arrhythmia to optimize prophylaxis and risk mitigation for a precision medicine approach.

Our study has some limitations. This model was trained on the Chapman training set which is not composed exclusively of critically ill patients. Despite this, the performance of the model on telemetry data of exclusively critically ill patients was very good. Model performance degraded slightly in the presence of background noise or artifact of the type that is sometimes seen in telemetry data due to patient movement, lead displacement, or bedside procedures. However, it still showed good performance when noisy data were included. Our use of a binary classifier resulted in the removal of 115 (12%) samples with non-AF and non-sinus rhythms, of which 87 were classified as non-diagnostic. While this is less than the > 25% loss using other noise detection and pre-processing methods, it does limit its use in a “real-time” clinical setting where the model would have to evaluate all rhythm strips. While 665 ECG samples were annotated by two reviewers, an additional 319 samples were annotated by a single reviewer. The agreement between reviewers was excellent and this was unlikely to contribute uncertainty to the model. Although it is possible to reduce the false positive rate by tuning the model decision threshold, doing so comes with the trade-off of decreased sensitivity. A future validation study to evaluate model performance in a prospective and continuous fashion is warranted. Further work is also needed to ‘clean’ raw data by removing artifacts.

## Conclusion

We adapted a deep learning model trained on static ECG data, to continuous telemetry data from an ICU in order to detect AF in critically ill patients. The model showed a sensitivity of 84%, a specificity of 89%, a PPV of 55%, and a NPV of 97%, allowing enhanced detection of AF beyond the 14% baseline prevalence of AF in the dataset. This model provides the foundation for enhanced detection and predictive model development to improve our understanding of the true burden of AF and its clinical consequences, and enhance classification and stratification of patients with NOAF for further studies.

## Supplementary Information

**Additional file 1:** **Figure S4.** Diagram of the neural network. **Table S3.** Specific categories of noise observed in the labeled KGH dataset during the noise annotation process. Segments not provided with a noise label are listed here as "No comment". **Figure S5.** Example segments for each noise category in the labeled KGH dataset. Only lead I is shown on these 10-s samples. **a** Clean atrial fibrillation **b** atrial fibrillation with baseline wander **c** normal sinus rhythm with baseline artifacts **d** non-diagnostic sample (annotator abstained). **Figure S6.** ROC curves for the deep learning model on clean, noisy, and all labeled sinus and AF samples from the KGH dataset

## Data Availability

The datasets used and/or analyzed during the current study are available from the corresponding author on reasonable request.
