# Supplementary material for: A deep learning model for the classification of atrial fibrillation in critically ill patients
Source: Intensive Care Med Exp. 2023 Jan 13;11:2. doi: 10.1186/s40635-022-00490-3 (PMC9837355; doi:10.1186/s40635-022-00490-3)
Supplement: Supplementary file 1 — Additional file 1: Figure S4. Diagram of the neural network. Table S3. Specific categories of noise observed in the labeled KGH dataset during the noise annotation process. Segments not provided with a noise label are listed here as "No comment". Figure S5. Example segments for each noise category in the labeled KGH dataset. Only lead I is shown on these 10-s samples. a Clean atrial fibrillation b atrial fibrillation with baseline wander c normal sinus rhythm with baseline artifacts d non-diagnostic sample (annotator abstained). Figure S6. ROC curves for the deep learning model on clean, noisy, and all labeled sinus and AF samples from the KGH dataset [file 40635_2022_490_MOESM1_ESM.docx]

**Additional file 1**

**Model Architecture and Training Details**

This deep convolutional neural network (CNN) comprised 13 blocks of 1D convolutions, batch normalization, ReLU activation and 30% dropout. We increased the number of input channels in the first convolutional layer from 1 to 4 so that the model could accept 4 channels (1 lead per channel) of ECG signal. Dilated max-pooling was selectively applied at blocks 6 and 11, while per-block dilation is gradually increased from 1 to 8. Global average pooling was used to accommodate variable-length inputs and condense the convolutional layer representations into a latent vector of 64 features.  A final linear layer and softmax activation was then applied to generate binary outputs for classification.

Unless otherwise specified, models were trained for a maximum of 200 epochs with early stopping after 10 epochs of no loss improvement. We used a batch size of 128, and the Adam optimizer with a learning rate of 0.01 and weight decay of 0.0001. For model selection, a grid search was conducted over the following hyperparameter ranges: batch sizes of 64 and 128, learning rates of 0.1, 0.01 and 0.001, weight decay of 0.01, 0.001 and 0.0001, and maximum epochs of 100 and 200.


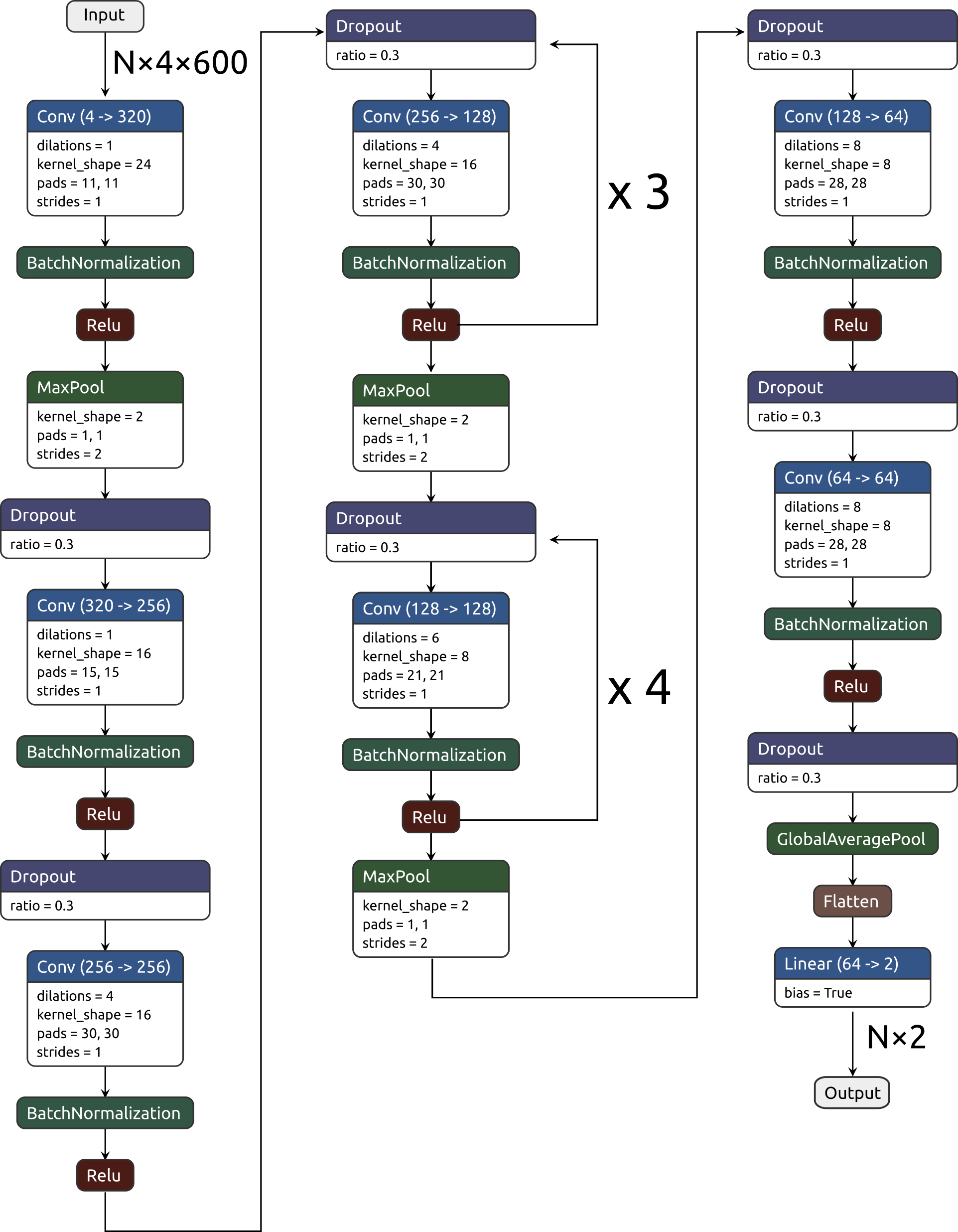


*Figure S4 – Diagram of the neural network*

**KGH Dataset Noise Characteristics**

| Description | Count |
| --- | --- |
| Baseline Artifact | 40 |
| Baseline Wander | 11 |
| Other Artifacts | 5 |
| No comment | 207 |

*Table S3. Specific categories of noise observed in the labelled KGH dataset during the noise annotation process. Segments not provided with a noise label are listed here as "No comment".*

| 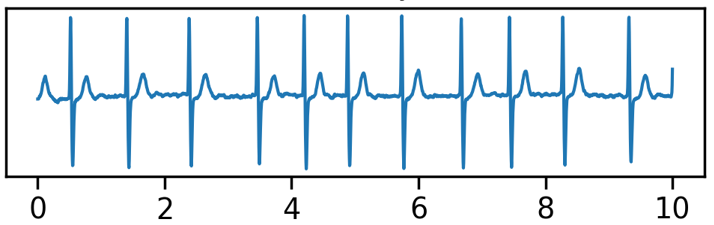(a) | 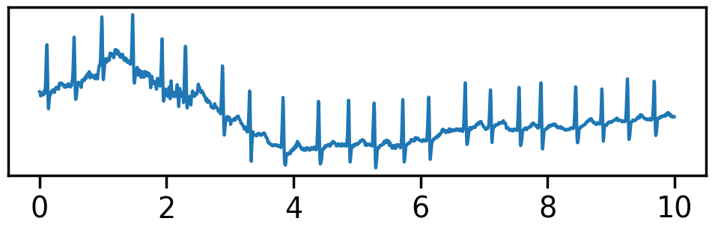(b) |
| --- | --- |
| 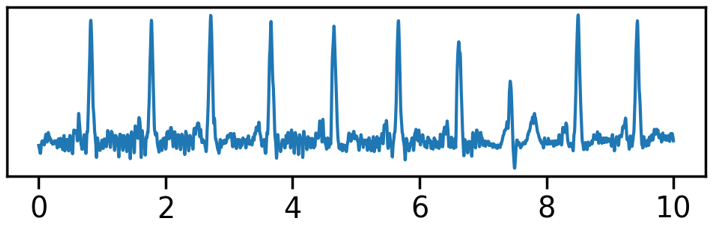(c) | 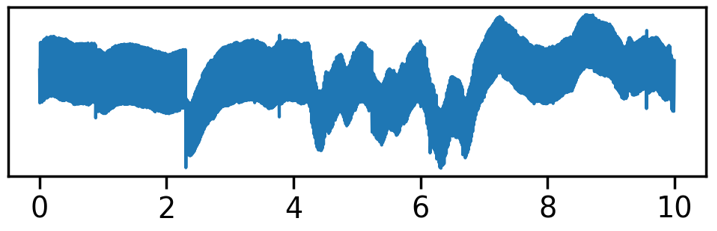(d) |

*Figure S5: Example segments for each noise category in the labelled KGH dataset. Only lead I is shown on these 10-second samples. (a) clean atrial fibrillation (b) atrial fibrillation with baseline wander (c) normal sinus rhythm with baseline artifacts (d) non-diagnostic sample (annotator abstained).*

**Extended Performance Results**


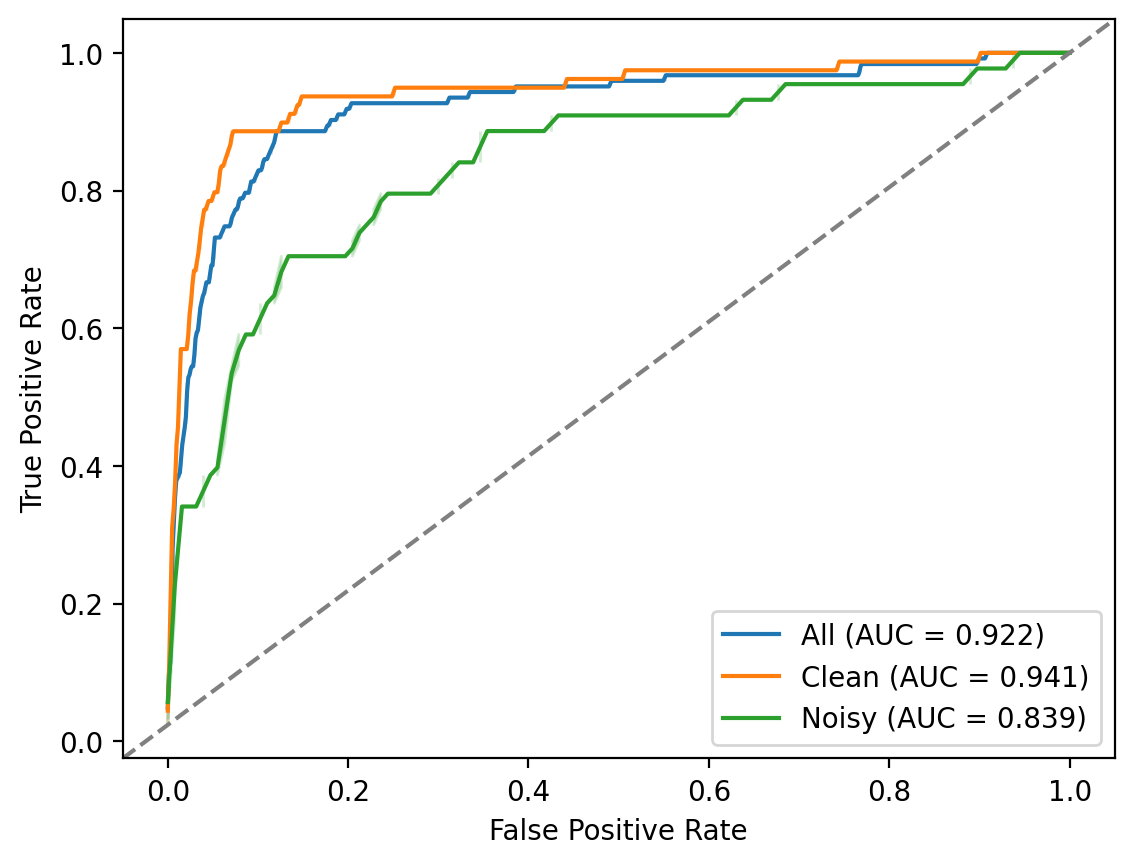


*Figure S6. ROC curves for the deep learning model on clean, noisy, and all labelled sinus and AF samples from the KGH dataset.*
